# HDAC6 Modulates Signaling Pathways Relevant to Synaptic Biology and Neuronal Differentiation in Human Stem Cell-Derived Neurons

**DOI:** 10.3390/ijms20071605

**Published:** 2019-03-31

**Authors:** Jonathan Iaconelli, Lucius Xuan, Rakesh Karmacharya

**Affiliations:** 1 Center for Genomic Medicine, Harvard Medical School and Massachusetts General Hospital, 185 Cambridge Street, Boston, MA 02114, USA; jiaconel@scripps.edu (J.I.); lxuan@partners.org (L.X.); 2 Chemical Biology Program, Broad Institute of Harvard and MIT, Cambridge, MA 02142, USA; 3 Schizophrenia and Bipolar Disorder Program, McLean Hospital, Belmont, MA 02478, USA; 4 Program in Neuroscience, Harvard University, Cambridge, MA 02138, USA; 5 Chemical Biology PhD Program, Harvard University, Cambridge, MA 02138, USA

**Keywords:** HDAC6, HDAC inhibitor, acetylation, β-catenin, AKT, synapse, neuronal differentiation

## Abstract

Recent studies show that histone deacetylase 6 (HDAC6) has important roles in the human brain, especially in the context of a number of nervous system disorders. Animal models of neurodevelopmental, neurodegenerative, and neuropsychiatric disorders show that HDAC6 modulates important biological processes relevant to disease biology. Pan-selective histone deacetylase (HDAC) inhibitors had been studied in animal behavioral assays and shown to induce synaptogenesis in rodent neuronal cultures. While most studies of HDACs in the nervous system have focused on class I HDACs located in the nucleus (e.g., HDACs 1,2,3), recent findings in rodent models suggest that the cytoplasmic class IIb HDAC, HDAC6, plays an important role in regulating mood-related behaviors. Human studies suggest a significant role for synaptic dysfunction in the prefrontal cortex (PFC) and hippocampus in depression. Studies of HDAC inhibitors (HDACi) in human neuronal cells show that HDAC6 inhibitors (HDAC6i) increase the acetylation of specific lysine residues in proteins involved in synaptogenesis. This has led to the hypothesis that HDAC6i may modulate synaptic biology not through effects on the acetylation of histones, but by regulating acetylation of non-histone proteins.

## 1. Introduction

**Figure 1:**
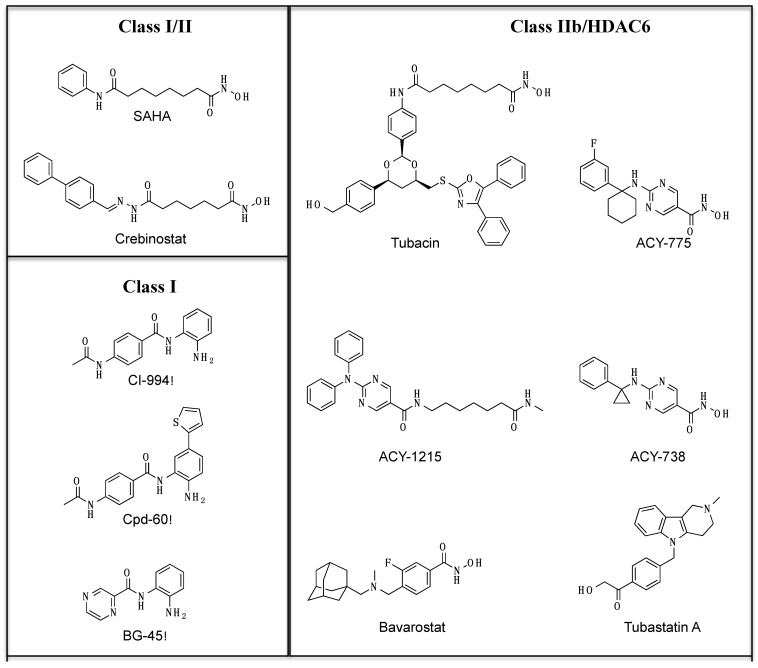
Structure of representative small molecules in the HDAC inhibitor toolkit.

Histone deacetylases (HDACs) and HATs (histone acetyltransferases) have been studied extensively for their role in regulating chromatin function through acetylation of histone proteins, including in neurons [1,2]. HDACs have important roles in learning and memory and in synaptic plasticity [3,4,5]. Different HDAC isoforms have been shown to control synapse maturation and function in mice with conditional alleles of HDAC isoforms and HDACi induce synaptogenesis in vitro in rodent neuronal cultures [6,7]. Many of these studies have focused on class I HDACs (HDACs 1,2,3) located in the nucleus and most HDACi studied to date have broad selectivity and target multiple HDAC isoforms [8]. However, there is a growing recognition of cellular processes where HDACs play crucial roles by deacetylating non-histone proteins [9]. The nature and extent of non-histone acetylation, especially in neurons, remain to be elucidated. Recent advances in chemical biology approaches have resulted in a better understanding of the functions of different HDAC isoforms and led to the development of small-molecule probes that target specific HDAC isoforms (Figure 1) [10]. For instance, SAHA and crebinostat target both class I and class II HDACs, CI-994, Cpd-60, and BG-45 are more specific for class I HDACs. Most HDAC inhibitors are designed with the framework of a pharmacophore model that includes a cap, a linker and a chelator. The chelator binds to the zinc ion at the HDAC active site, while the linker connects this chelator with the capping motif that engages the outer edge of the active site region [10]. Efforts to design isoform-selective small molecule inhibitors have focused on employing variations in the cap and chelator functions. While many HDAC inhibitors are designed for better isoform selectivity, many of the inhibitors do inhibit other HDACs as well at higher doses. Since the original discovery of tubacin as a HDAC6 inhibitor, there have been a number of new small molecules that have been synthesized that show better isoform selectivity for HDAC6, including some with brain bio-availability [11,12,13,14,15,16,17,18,19,20,21,22] (Table 1). For the HDAC6 inhibitor ACY-738, mice treated for 21 days resulted in a brain–plasma ratio of ~1.4 while treatment for 90 days showed brain–plasma ratio of ~2.3 [23]. The brain bio-availability of bavarostat was shown through the radiochemical synthesis of [18] F bavarostat and the demonstration of uptake and retention of the compound in the rat brain with pet imaging [20].

## 2. Role of HDAC6 in Animal Models Relevant to Nervous System Disorders

Studies in the last few years have led to discoveries on the role of HDAC6 in biological processes implicated in a number of neurodegenerative, neurodevelopmental, and neuropsychiatric disorders. One of these areas relates to neuronal injury and regeneration in the context of axonal degeneration from excitotoxicity. In mouse cortical neuron cultures, it was shown that inhibiting HDAC6 through genetic and pharmacological methods led to increased survival and neuronal regeneration in the setting of oxidative stress [24]. In an another study using mouse cortical neuron cultures, it was shown that axonal degeneration from kainic acid was accompanied by significant decrease in α-tubulin acetylation [25]. Treatment with an HDAC inhibitor resulted in higher levels of α-tubulin acetylation, as expected, but it also protected the neurons from kainic acid-induced axonal degeneration [25].

Studies examining the biology of amyotrophic lateral sclerosis (ALS) have led to a focus on HDAC6 as a putative therapeutic target [26]. A number of ALS-associated proteins have been found to regulate the activity of HDAC6, specifically the proteins Fused in Sarcoma/Translocated in Sarcoma (FUS/TLS) and TAR DNA binding protein-43 (TDP-43) [27,28,29,30]. Some of the early studies suggested that HDAC6 may have a protective effect in ALS, and it was found SOD1^G93A^ mice has lower levels of HDAC6 expression at disease onset which became very low at the later stages [31]. In cultured cells, it was initially shown that HDAC6 knockdown led to increased aggregation of mutant SOD1 and that HDAC6 may facilitate degradation of aggregation-prone proteins in the cytoplasm [32,33]. However, in the SOD1^G93A^ mouse model of ALS, deletion of the HDAC6 gene led to a significant decrease in progression of disease and resulted in prolonged survival, which was accompanied by increased α-tubulin acetylation [34]. Given the contradictory nature of those studies, the role of HDAC6 in ALS is unclear. These studies relied on genetic methods of abolishing HDAC6 function. While small-molecule studies complement genetic loss-of-function studies, the correlation between genetic loss of function and small-molecule inhibition profiles is not always consistent with each other [35,36]. It would be informative to examine how pharmacological inhibition of HDAC6 would alter disease progression in these animal models.

A number of animal studies have also pointed to a role for HDAC6 in Alzheimer’s disease. In an amyloid precursor protein/presenilin 1 (APP/PS1) mouse model, treatment with the HDAC6 inhibitor ACY-738 resulted in improvement of axonal transport rates, decrease in phosphorylation of tau and increase in α-tubulin acetylation, accompanied by improvement in amyloid pathology as well as contextual learning and memory [23]. Studies in mice and rodent neurons showed that genetic or pharmacological reduction in HDAC6 activity can also decrease tau mislocalization and suppress the pathogenic formation of neuritic tau beads [37]. When APPPS1-21 mice, which show memory impairment, amyloid pathology and low levels of α-tubulin acetylation, were crossed with *Hdac6* knockout mice, it was found that the reduction in HDAC6 led to improvement in memory function, accompanied by robust increases in acetylated α-tubulin [38]. In a study using rTg4510 mouse model of tau deposition, it was shown that treatment with the HDAC6 inhibitor Tubastatin A resulted in improved memory function as well as decreased levels of tau [39]. To confirm that these effects are due to specific inhibition of HDAC6 by Tubastatin A, Tg4510 mice can be crossed with Hdac6 knockout mice in order to examine the effects on memory formation and tau levels, In another study of an Alzheimer’s disease mouse model treatment with ACY-1215 and Tubastatin A, both led to improvement in the behavioral assays as well as changes in amyloid β levels, decrease in phosphorylation of tau and increase in α-tubulin acetylation [40].

The Alzheimer’s disease mouse model harboring APP^Swe^ and tau^P301L^ mutant transgenes develops both tangles and plaques and shows impairment in learning and memory tasks [41]. Pharmacological inhibition of HDAC6 in these mice led to improvement in learning and memory tasks, accompanied by increased α-tubulin acetylation in the brain as well as decreased tau S396 and S404 phosphorylation [42]. Experiments in SH-SY5Y and Neuro2a cell lines showed that pharmacological HDAC6 inhibition resulted in reduced phosphorylation and aggregation of tau and increased Hsp90 acetylation, accompanied by increased phosphorylation by Akt of the S9 residue on glycogen synthase kinase β (GSK3β) [42].

Recent studies of Charcot-Marie-Tooth disease suggest that HDAC6 may be a promising target for this disorder as well [43,44,45]. Cultured DRG neurons from a mutant HSPB1 mouse model of Charcot-Marie-Tooth disease showed deficits in axonal transport and this deficit was rescued by the HDAC6 inhibitors ACY-738 and ACY-775 [46]. Another mouse model of Charcot-Marie-Tooth disease, based on dominant mutations in glycyl-tRNA synthetase, showed that the mice have aberrant axonal transport and this is accompanied by decreased α-tubulin acetylation [47]. Treatment with the HDAC6 inhibitor Tubastatin A led to increased α-tubulin acetylation, ameliorated the deficits in axonal transport and improved the motor functioning in the mutant mice [47]. In a study of cortical neurons from a Rett syndrome MECP2^T158A^ mouse model and of patient fibroblasts, it was found that the cortical neurons of the MECP2-deficient mice and the patient fibroblasts had increased levels of HDAC6 protein expression and reduced levels of acetylated α-tubulin and treatment with Tubastatin A resulted in increased levels of acetylated α-tubulin [48].

In addition to the role of HDAC6 in neurons, animal studies show a role for HDAC6 in oligodendrocytes as well [49,50]. Cultured rat oligodendrocytes were shown to express HDAC6 and inhibition of HDAC6 by Tubastatin A resulted in decreased microtubule binding activity of tau [51]. It was shown that HDAC6 inhibition led to increased acetylation of tau in the oligodendrocytes, which in turn reduced its turnover rate [51]. Furthermore, proteasomal inhibition led to the accumulation of acetylated tau and HDAC6 in protein aggregates, which was altered by Tubastatin A or RNAi-mediated downregulation of HDAC6 [52]. In addition, experiments in oligodendroglial cell lines showed that HDAC6 dysregulation played a role in stress responses in these cells [52].

HDAC6 has also been implicated in animal models of retinal diseases that involve loss of photoreceptors. It has been hypothesized that HDAC6 has a role in protecting photoreceptors and retinal cells that are vulnerable to reactive oxygen species from oxidative stress-related damage [53]. HDAC6 is constitutively expressed in the retina in mice and in the cone-like rodent cell line 661W [53]. Inhibition of HDAC6 by Tubastatin A upregulated heat-shock proteins HSP25 and HSP70 and led to increased cell survival in the setting of oxidative stress [53]. Similarly, in a zebrafish model of inherited sight loss, in vivo treatment with Tubastatin A ameliorated morphological features in the retina and improved visual function [53].

## 3. Studying HDAC Biology in Human Neuronal Cells

Studies in rodents have yielded a wealth of knowledge on basic biology and pathophysiology, including in our understanding of neurobiology and animal models have been routinely used to identify new therapeutic leads for various human diseases, including for psychiatric disorders [54,55]. While these studies have led to a better understanding of the biology, there has been a dearth of compounds that have translated successfully from animal models to humans [56,57,58,59,60]. Studies show that genomic responses in humans to specific pathophysiological processes often have poor correlation with such responses in rodent models [61]. Recent studies in induced human neurons and in cortical neurons from knockout mice showed species-specific differences in the effects of NRXN1 mutations on synaptic biology [62]. When heterozygous conditional loss-of-function *NRXN1* mutations were introduced into human embryonic stem cells (ESCs), it resulted in severe deficits in stimulus-dependent transmitter release but similar experiments in mice with Nrxn1α mutations did not show this impairment [62]. These considerations have led to a note of caution about focusing exclusively on tissue from animal studies for preclinical investigations and have led to the development of different approaches to study the biology of disease and therapeutics in cells from human subjects [63,64,65,66,67].

Cellular reprogramming methods enable generation of human induced pluripotent stem cells (iPSCs) [68,69]. Human iPSCs can be differentiated along neuronal lineages both to study disease biology and to identify new therapeutic leads [70,71,72,73]. Given the complexity of cortical development and the diversity of neurons, generation and identification of specific neuronal subtypes seem daunting. However, there have been recent methodological advances in human iPSC differentiation along specific neuronal lineages [74]. Human iPSCs can be differentiated to cortical neurons that express markers for different cortical layer neurons [72,74]. This enables the dissection of the phenotypic effects of small molecules in specific neuronal subtypes.

Ex vivo cellular models of disease based on patient-derived iPSCs provide powerful approaches to dissecting the molecular underpinnings of disease biology and complement/confirm findings from rodent models of different diseases. In a study conducted with iPSCs from Charcot-Marie-Tooth disease 2F (CMT2F) and distal hereditary motor neuropathy 2B (dHMN2B) carrying heat shock 27 kDa protein 1 (HSPB1), it was found that the motor neurons differentiated from the mutant lines showed decreased acetylation of α-tubulin and significant deficits in the axonal movement of mitochondria [75]. Treatment with two different pharmacological inhibitors of HDAC6 led to increased acetylation of *α*-tubulin and rescue of the deficits in movement of mitochondria in the axons [75].

A recent study compared iPSC-derived motor neurons from ALS patients with FUS mutations with motor neurons from healthy control subjects along with isogenic lines generated using CRISPR-Cas9 [76]. Motor neurons differentiated from FUS3 mutant iPSC lines, as well as motor neurons from human embryonic stem cells (hESCs) expressing mutant FUS, showed characteristic cellular and functional findings of cytoplasmic FUS accumulation, hypoexcitability, and deficits in axonal transport [76]. They further showed that inhibiting HDAC6, with the HDAC6-specific inhibitors Tubastatin A and ACY-738 or with antisense oligonucleotides, rescued the deficits in axonal transport in those motor neurons [76].

Cellular models using iPSC-derived neurons have also been used to study the role of HDAC6 in Rett syndrome [77]. Neurons differentiated from iPSCs of Rett syndrome patients with MECP2 mutations showed a transcriptomic profile that indicated disruption in GABAergic circuits as well a significant upregulation of HDAC6 [77]. This was accompanied by decreased levels α-tubulin acetylation in the MECP2 neurons, which were reversed with treatment with the HDAC6 inhibitor ACY-1215 [77].

The results of studies examining HDAC6 function in animal models and in human neuronal cells are summarized in Table 2.

## 4. HDACs and Wnt-GSK3-β-Catenin Biology in Mood Disorders

HDACs are hypothesized to be important in the biology of mood disorders based on mRNA expression of HDAC isoforms in patients with mood disorders and modulation of their expression by antidepressants and mood stabilizers [8,78,79,80]. The mood stabilizer valproic acid has been shown to possess HDAC inhibitory activity [81]. Multiple HDACi have antidepressant-like activity in pre-clinical animal models, including sodium butyrate, suberoylanilide hydroxamic acid (SAHA), MS-275, and Cpd-60 [82,83,84,85,86]. In mice, the SSRI fluoxetine and the class I HDACi MS-275 reverse the effects of chronic social defeat stress on global patterns of gene expression [8]. It has been shown that the HDACi Cpd-60 modulates mood-related behaviors in mice and induces brain gene-expression changes that overlap with lithium treatment [83]. Recently, brain-penetrant HDAC6-selective inhibitors were shown to induce antidepressant-like behavior in the tail suspension test and the social defeat paradigm in mice [22]. Antidepressant-like behaviors were also described in rodents with loss of function of HDAC6 [22,87].

The molecular mechanisms through which HDAC6i leads to antidepressant-like behavior remain poorly understood, although consistent with a role for non-histone substrates. Much of our knowledge of the biology of HDAC6 and the Wnt-GSK3-β-catenin pathway comes from studies in rodents. Studies aimed at understanding the synaptogenic effects of HDACi with a focus on β-catenin are being explored since β-catenin plays an important role in synaptic plasticity [88]. Studies suggest that β-catenin is a key component in the biology of mood disorders and in the therapeutic effects of antidepressants and mood stabilizers [89,90,91,92,93]. Transgenic mice that express a constitutively active form of β-catenin in the adult brain show antidepressant-like effects [89]. Conversely, mice with forebrain-specific β-catenin knockout exhibit depressive-like behavior [90]. The function and stability of β-catenin is modulated by GSK3β. GSK3β phosphorylates β-catenin and primes it for proteasomal degradation. Postmortem studies in unipolar and bipolar depression show decreased activation of GSK3β in patient brains [94,95]. The mood stabilizer lithium inhibits GSK3β and this inhibition is hypothesized to mediate its therapeutic efficacy [91,96]. In rodents, lithium shows dose-dependent antidepressant-like effects [97,98]. While homozygous GSK3β-/- mice die in utero, heterozygous loss of GSK3β mimics lithium’s antidepressant-like effects [99].

β-catenin, an integral component of the Wnt signaling pathway, is an evolutionarily well-conserved protein with pivotal roles in the developing and adult central nervous system [100,101,102]. When the Wnt pathway is inactive, β-catenin is retained in the cytoplasm in a “destruction complex” with Axin, adenomatosis polyposis coli (APC), and GSK3β [102]. In this complex, GSK3β constitutively phosphorylates β-catenin. This phosphorylated version of β-catenin is recognized by βTrCP of the E3 ubiquitin ligase complex and degraded by the proteasome [102]. When Wnt ligands bind to the Frizzled/LRP receptor, Axin is recruited to the membrane, resulting in dismantling of the destruction complex. β-catenin then accumulates and translocates to the nucleus, where it binds to the T-cell Factor (TCF) transcription factors and increases transcription of target genes [102].

## 5. Effect of HDAC6 Inhibition on β-Catenin Biology in Human Neuronal Cells

A set of well-annotated HDACi tool compounds (Figure 1) have been used to examine the effects of different HDACs on acetylation of β-catenin in human iPSC-derived neural progenitor cells (NPCs) [103]. The lysine residue K49 on β-catenin is a post-translational modification (PTM) site that is known to be acetylated [104]. K49 is adjacent to the phosphorylation sites for GSK3β and Casein Kinase 1α (CK1α) in the N-terminal regulatory domain. K49 is often found mutated to arginine in anaplastic thyroid carcinomas, resulting in increased nuclear localization of β-catenin [105]. In cancer cell lines SW480 and HCT116, K49 deacetylation was necessary for epidermal growth factor (EGF)-induced nuclear localization of β-catenin [106]. However, outside of these tumor cell lines, the function and regulation of this K49-β-catenin acetylation remain poorly understood. The isoform-selective HDACis, especially ones that target HDAC6, have enabled recent studies on the role of K49 acetylation on β-catenin in human neurons.

While human NPCs had minimal Ac-K49-β-catenin at baseline, treatment with the pan-HDACi SAHA and another broadly selective HDACi crebinostat led to a marked increase in Ac-K49-β-catenin levels, without affecting overall β-catenin levels [103]. Class I specific HDACi MS-275 and BG-45 did not increase Ac-K49-β-catenin, but treatment with HDAC6i ACY-1215 resulted in marked increase in Ac-K49-β-catenin. Experiments were undertaken to investigate how K49 acetylation on β-catenin affected adjacent phosphorylation sites in the N-terminal regulatory domain—CK1α site S45 and GSK3β sites S33/S37/T41. There was minimal S45 phosphorylation at baseline but increased K49 acetylation with HDAC6i was mirrored by increased S45 phosphorylation without any impact on phosphorylation of S33/S37/T41 [103]. While CK1α phosphorylates S45, the functional consequence of this phosphorylation is not understood [107]. There have been different hypotheses about the interaction between those posttranslational modifications. In one model, S45 phosphorylation primes β-catenin for GSK3β phosphorylation at S33/S37/T41 [108]. Another model posits that S45 phosphorylation is uncoupled from S33/S37/T41 phosphorylation [109]. Our results support the second model since HDAC6i-induced K49 acetylation led to increased phosphorylation of S45 but not of S33/S37/T41.

K49 is a known site for ubiquitination, which primes β-catenin for degradation by the proteasome [110]. There is an increasing recognition of the crosstalk between acetylation and ubiquitination of lysine residues in a number of different proteins [111,112,113,114]. There have been suggestions of crosstalk between acetylation and ubiquitination on β-catenin as well [115]. To explore this, the effect of the proteasome inhibitor MG-132 in human NPCs was studied in the presence or absence of HDAC6 inhibition. In NPCs treated with MG-132, there was significant accumulation of ubiquitinated β-catenin, along with increase in total β-catenin [103]. However, in NPCs pre-treated with the HDAC6 inhibitor ACY-1215, there was minimal change in ubiquitinated β-catenin or total β-catenin in the presence of MG-132 [103]. Total ubiquitination levels were unaffected by ACY-1215 pre-treatment, which indicated that ACY-1215 effects on ubiquitination were specific for β-catenin. These findings indicate that HDAC6i-mediated K49 acetylation on β-catenin has an impact on its ubiquitination.

Since acetylation can mediate subcellular localization of non-histone proteins [9], experiments were undertaken to examine whether increased K49 acetylation was accompanied by changes in subcellular localization of β-catenin. Immunofluorescence experiments showed that that exposure to ACY-1215 resulted in increased β-catenin at the plasma membrane in human NPCs, where it co-localized with N-cadherin [103]. Cell fractionation studies of NPCs treated with ACY-1215 showed that the increase in Ac-K49-β-catenin was in the membrane/cytoplasmic fraction and not in the nuclear fraction. With N-cadherin immunoprecipitation in NPCs, ACY-1215 resulted in increased levels of Ac-K49-β-catenin bound to N-cadherin [103]. To corroborate the small-molecule findings, siRNA knockdown of HDAC6 in the human NPCs were undertaken as well. HDAC6 knockdown in the human NPCs resulted in increased Ac-K49 and p-S45-β-catenin levels, similar in magnitude as with ACY-1215, without any change in total β-catenin [103]. HDAC6 knockdown in human NPCs also led to increase in β-catenin at the plasma membrane [103].

## 6. β-Catenin’s Function at the Synapse

In addition to the central role in the Wnt signaling pathway, β-catenin also forms a complex with cadherins at the membrane as part of the adherens junctions, including with N-cadherin in neuronal cells [88,100,116]. The β-catenin/N-cadherin complex is important for cell-cell adhesion in the nervous system and is present in both pre and postsynaptic neurons [88,100,116]. The N-cadherin/β-catenin complex on dendrites and axons form extracellular interactions between the cadherins while β-catenin links with the actin cytoskeleton. Extracellular homophilic interactions between N-cadherins and the intracellular interactions of β-catenin stabilize the axonal and dendritic contacts to facilitate synapse formation [116].

β-catenin modulates multiple aspects of synaptic biology, including spine structure, dendritic arborization, and synaptic plasticity [116]. Loss of GSK3β in the cortex and hippocampus in adult mice has been shown to affect spine density and synaptic stabilization through effects on β-catenin [117]. Maturation of dendritic spines in rodent brain is coordinated by β-catenin-N-cadherin interactions and loss of β-catenin in glutamatergic neurons in rodent models has been shown to significantly alter synaptic structure and function [118]. Overexpression of β-catenin in vivo in mice showed increased dendritic growth and promoted neuronal activity [119]. The specific mechanisms behind how β-catenin modulates synaptic function remain to be elucidated [116].

It is possible that β-catenin-facilitated effects on synaptogenesis may mediate antidepressant activity of seemingly diverse therapeutic modalities. In mice, ketamine induced inhibitory serine phosphorylation of GSK3β, which was necessary for its antidepressant-like effects [120]. In rats, GSK3β inhibition potentiated antidepressant-like effects of sub-threshold doses of ketamine and increased spine density in mPFC pyramidal cells [121]. The antidepressant-like effects of 5-HT_2A_ receptor antagonist ketanserin and the SSRI fluoxetine in rats were accompanied by increase in the membrane fraction of β-catenin and a parallel increase in N-cadherin, but without any change in levels of nuclear β-catenin [122]. Electroconvulsive seizures (ECS) in rats showed up-regulation of β-catenin in the hippocampus [123]. Alternative mechanisms of β-catenin membrane stabilization that do not involve GSK3β are not well understood. These findings have led to the hypothesis that HDAC6i effects on β-catenin may mediate enhanced synaptogenesis and antidepressant effects.

## 7. Implications for HDAC6i Mediated β-catenin Membrane Localization

**Figure 2:**
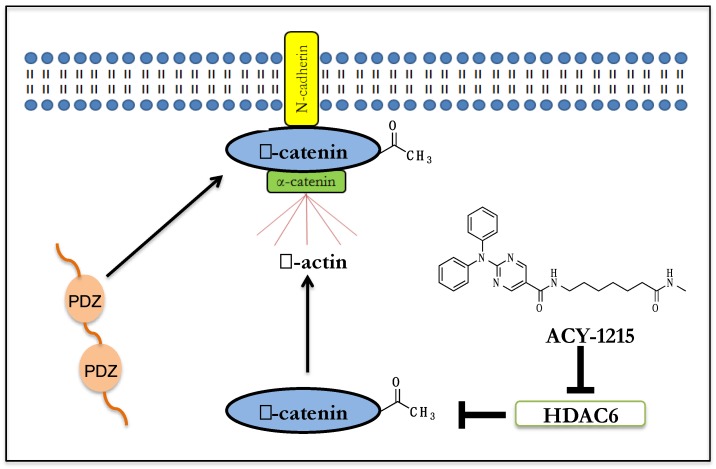
Schematic model of putative HDAC6i effect in human neurons: HDAC6i increases K49-β-catenin acetylation, which results in membrane localization of β-catenin. β-catenin binds to N-cadherin and recruits PDZ-containing proteins involved in synaptic stabilization.

β-catenin links N-cadherin to the actin cytoskeleton and recruits intracellular partners to the synaptic membrane to form co-complexes with N-cadherin [116]. Studies in human NPCs had shown that HDAC6 inhibition results in the translocation and enrichment of β-catenin to the plasma membrane [103]. β-catenin has a PDZ-binding domain which has been shown in a protein microarray study to bind to over two-dozen proteins containing PDZ domains [116]. β-catenin has also been shown to co-localize with a number of the PDZ-domain-containing proteins at the plasma membrane [116]. Synapses are enriched for a diverse array of proteins with multiple PDZ domains, including ones involved in synaptic stabilization. PDZ-domain-containing proteins often act as intracellular scaffolds at synapses to co-localize different proteins and facilitate signaling [124,125,126]. β-catenin regulates synaptic vesicle localization by recruiting synaptic proteins with PDZ domains [116]. β-catenin has been shown to physically bind to a number of synaptic proteins with PDZ domains [127]. These studies suggest a role for β-catenin in HDAC6i effects on synaptogenesis through the interaction of acetylated β-catenin to PDZ-domain-containing synaptic proteins (Figure 2).

## 8. HDAC6 Interaction with AKT and Relevance to Neurobiology

In addition to β-catenin, HDAC6 deacetylates lysine residues on α-tubulin, HSP90, and AKT [16,128,129]. A number of antipsychotic medications and mood stabilizers are hypothesized to mediate their effects through modulation of the PI3K-AKT-GSK3 pathway [130,131,132]. AKT knockdown also adversely impacts synaptogenesis in rodent hippocampal neurons, accompanied by decreased dendritic spine density [133]. Human neuronal cells treated with HDAC6i show increased AKT acetylation, specifically at residues K163 and K377 in the kinase domain [128], which are distinct from the PH-domain sites deacetylated by the class III KDACs SIRT1 and SIRT2 [134,135]. Treatment with HDAC6i led to reduced binding of AKT to PIP_3_ and decreased its ability to phosphorylate downstream targets including S552 on β-catenin that mediates its subcellular localization [128]. Hence, HDAC6 may also modulate synaptic function through modulating the acetylation status of K163 and K377 on AKT.

Studies of cortical development in mice had shown that decreased AKT activity in NPCs during development affected neuronal differentiation [136]. AKT has important roles during neuronal differentiation, and p-AKT(Ser^473^), which is active, is present in NPCs in the cortex [136,137]. Studies with the HDAC6 inhibitors in human iPSC-derived NPCs showed that HDAC6 inhibition did not have any effects on NPC proliferation per se. However, exposure to HDAC6 inhibition during the neuronal differentiation process led to the generation of neuronal cultures which had a greater proportion of glial cells in relation to neuronal cells. These results suggest that HDAC6 and class I HDACs have opposite effects during neuronal differentiation since inhibition of class I HDACs promote differentiation along the neuronal lineage while HDAC6 inhibition led to the generation of greater proportion of glial cells [128,138].

## 9. Summary

Recent studies in animal models as well as in human neurons have led to a better understanding of the role of HDAC6 in cellular processes that have important roles in the biology of various neurodevelopmental, neurodegenerative, and neuropsychiatric disorders. While early studies of HDAC6 were hindered by the lack of small molecules with good isoforms selectivity, the development of isoform-specific inhibitors in the last few years have enabled incisive chemical biology studies aimed at interrogating the role of HDAC6 in different cellular contexts, including in neuronal differentiation and studies of synaptic biology. Studies of HDAC6 inhibitors in human neuronal cells have shown that HDAC6i modulate the acetylation of specific lysine residues on β-catenin and AKT, two proteins with known roles in synaptic biology. HDAC6 inhibition results in increased acetylation of K49 on β-catenin and increased accumulation of β-catenin at the plasma membrane. At the synapse, β-catenin links N-cadherin to the actin and recruits intracellular partners to the synaptic membrane. Since β-catenin has a PDZ-binding domain that binds PDZ-domain-containing synaptic proteins, HDAC6i may enhance synaptogenesis and synaptic stabilization by modulating the enrichment of acetylated β-catenin at the synapse. These findings raise the possibility that HDAC6i may modulate synaptic biology by regulating acetylation of non-histone proteins, which can be leveraged to in the development of novel therapeutic leads aimed at modulating synaptic biology. Though clinical trials of HDAC6 inhibitors to date have primarily focused on cancer therapies, there is growing evidence that HDAC6 inhibitors may have clinical applications in neuropsychiatric disorders as well. The introduction of HDAC6 inhibitors in these disorders will need a careful assessment not only of the scientific rationale and the preclinical data that points to efficacy but also of the need for brain bio-available small-molecule candidates and careful consideration of any possibility of neurotoxicity.

## Figures and Tables

**Table 1:** IC50 values (µM) for inhibition of different HDAC isoforms.

	HDAC1	HDAC2	HDAC3	HDAC4	HDAC5	HDAC6	HDAC7	HDAC8	HDAC9	Selectivity	Reference
SAHA	0.0013	0.0016	0.005	_	3.6	0.0016	_	0.48	_	1,2,3,6,8	[11]
Crebinostat	0.0007	0.001	0.002	_	_	0.009	_	_	_	1,2,3,6	[12]
CI-994	0.05	0.19	0.55	_	_	_	_	_	_	1,2,3	[13]
Cpd-60	0.001	0.008	0.458	_	_	_	_	_	_	1,2	[14]
BG-45	2	2.2	0.289	_	_	>20	_	_	_	3	[15]
Tubacin	0.028	0.042	0.275	17	1.5	0.016	8.5	0.17	_	6	[16]
Tubastatin A	3.2	3.5	4.9	_	_	0.018	_	_	_	6	[17]
ACY-738	0.094	0.128	0.218	_	_	0.0017	_	_	_	6	[18]
ACY-775	2.123	2.57	1.12	_	_	0.0075	_	_	_	6	[18]
ACY-1215	0.058	0.048	0.051	7	5	0.004	1.4	0.1	10	6	[19]
Bavarostat	>1000	>1000	>1000	11.3	19	0.06	4.7	8.5	5.2	6	[20]
PCI-34051	>50	>50	6.8	_	_	2.9	_	0.01	>50	8	[21]

**Table 2:** Summary of studies examining effects of HDAC6 inhibition in the nervous system.

Study Model	HDAC6 Modulation	Effect of HDAC6 Inhibition	Citation
Mouse cortical neurons	Genetic and pharmacological (Trichostatin A)	Increased survival and regeneration in setting of oxidative stress.	Rivieccio et al. (2009) [24]
Mouse cortical neurons	Trichostatin A Scriptaid	Protection of kainic acid-induced axonal degeneration.	Hanson et al. (2018) [25]
SOD1^G93A^ ALS mouse model	HDAC6 deletion	Buildup of SOD1^G93A^ aggregates but only mild effects on motor function.	Lee et al. (2015) [33]
SOD1^G93A^ ALS mouse model	HDAC6 deletion	Decrease in disease progression and prolonged survival.	Taes et al. (2013) [34]
APP/PS1 mouse model	HDAC6 deletion	Improvement in memory function	Govindarajan et al. (2013) [38]
rTg4510 mouse model	Tubastatin A	Improvement in memory function and lower tau levels	Selenica et al. (2014) [39]
AD mouse model	Tubastatin A ACY-1215	Improvement in behavior and decrease in amyloid β and hyperphosphorylated tau.	Zhang et al. (2014) [40]
AD mouse model	MPT0G211	Improvement in learning and memory and decrease in tau phosphorylation.	Fan et al. (2018) [42]
Charcot-Marie-Tooth HSPB1 mouse model	ACY-738 ACY-775	Rescue of axonal transport deficits	Benoy et al. (2017) [43]
Charcot-Marie-Tooth GARS mouse model	Tubastatin A	Improved deficits in axonal transport & motor functioning	Shen et al. (2016) [44]
Cortical neurons from MECP2^T158A^ mouse model	Tubastatin A	Increased α-tubulin acetylation	Gold et al. (2015) [48]
Rett syndrome patient fibroblast	Tubastatin A	Ameliorated microtubule defects	Gold et al. (2015) [48]
Cultured rat oligodendrocytes	Tubastatin A shRNA	Reduced microtubule binding activity of tau. Reduced protein aggregation.	Noack et al. (2014) [51] Leyk et al. (2015) [52]
dye^ucd6^ zebrafish model	Tubastatin A	Rescued visual function and retinal morphology	Leyk et al. (2017) [53]
Motor neurons from iPSCs of CMT2F and dHMN2B patients	CHEMICAL X4 CHEMICAL X9	Reversed axonal movement defects of mitochondria	Kim et al. (2015) [75]
Motor neurons from iPSCs of FUS-ALS patients	ACY-738 Tubastatin A Antisense oligos	Restore axonal transport defects and increase mitochondria-ER overlay	Guo et al. (2017) [76]
Neurons from iPSCs of Rett syndrome patients with MECP2 mutations	ACY-1215	Reversal of decrease in α-tubulin acetylation	Landucci et al. (2018) [77]

## References

[1] Brownell J.E., Allis C.D. (1996). Special HATs for special occasions: Linking histone acetylation to chromatin assembly and gene activation. Curr. Opin. Genet. Dev..

[2] Hassig C.A., Schreiber S.L. (1997). Nuclear histone acetylases and deacetylases and transcriptional regulation: HATs off to HDACs. Curr. Opin. Chem. Biol..

[3] Cho Y., Cavalli V. (2014). HDAC signaling in neuronal development and axon regeneration. Curr. Opin. Neurobiol..

[4] Mahgoub M., Monteggia L.M. (2014). A role for histone deacetylases in the cellular and behavioral mechanisms underlying learning and memory. Learn. Mem..

[5] Fass D.M., Schroeder F.A., Perlis R.H., Haggarty S.J. (2014). Epigenetic mechanisms in mood disorders: Targeting neuroplasticity. Neuroscience.

[6] Shi P., Scott M.A., Ghosh B., Wan D., Wissner-Gross Z., Mazitschek R., Haggarty S.J., Yanik M.F. (2011). Synapse microarray identification of small molecules that enhance synaptogenesis. Nat. Commun..

[7] Rumbaugh G., Sillivan S.E., Ozkan E.D., Rojas C.S., Hubbs C.R., Aceti M., Kilgore M., Kudugunti S., Puthanveettil S.V., Sweatt J.D. (2015). Pharmacological Selectivity Within Class I Histone Deacetylases Predicts Effects on Synaptic Function and Memory Rescue. Neuropsychopharmacology.

[8] Covington H.E., Maze I., LaPlant Q.C., Vialou V.F., Ohnishi Y.N., Berton O., Fass D.M., Renthal W., Rush A.J., Wu E.Y. (2009). Antidepressant actions of histone deacetylase inhibitors. J. Neurosci..

[9] Choudhary C., Kumar C., Gnad F., Nielsen M.L., Rehman M., Walther T.C., Olsen J.V., Mann M. (2009). Lysine acetylation targets protein complexes and co-regulates major cellular functions. Science.

[10] Bradner J.E., West N., Grachan M.L., Greenberg E.F., Haggarty S.J., Warnow T., Mazitschek R. (2010). Chemical phylogenetics of histone deacetylases. Nat. Chem. Biol..

[11] Richon V.M., Webb Y., Merger R., Sheppard T., Jursic B., Ngo L., Civoli F., Breslow R., Rifkind R.A., Marks P.A. (1996). Second generation hybrid polar compounds are potent inducers of transformed cell differentiation. Proc. Natl. Acad. Sci. USA.

[12] Fass D.M., Reis S.A., Ghosh B., Hennig K.M., Joseph N.F., Zhao W.N., Nieland T.J., Guan J.S., Kuhnle C.E., Tang W. (2013). Crebinostat: A novel cognitive enhancer that inhibits histone deacetylase activity and modulates chromatin-mediated neuroplasticity. Neuropharmacology.

[13] el-Beltagi H.M., Martens A.C., Lelieveld P., Haroun E.A., Hagenbeek A. (1993). Acetyldinaline: A new oral cytostatic drug with impressive differential activity against leukemic cells and normal stem cells—Preclinical studies in a relevant rat model for human acute myelocytic leukemia. Cancer Res..

[14] Moradei O.M., Mallais T.C., Frechette S., Paquin I., Tessier P.E., Leit S.M., Fournel M., Bonfils C., Trachy-Bourget M.C., Liu J. (2007). Novel aminophenyl benzamide-type histone deacetylase inhibitors with enhanced potency and selectivity. J. Med. Chem..

[15] Minami J., Suzuki R., Mazitschek R., Gorgun G., Ghosh B., Cirstea D., Hu Y., Mimura N., Ohguchi H., Cottini F. (2014). Histone deacetylase 3 as a novel therapeutic target in multiple myeloma. Leukemia.

[16] Haggarty S.J., Koeller K.M., Wong J.C., Grozinger C.M., Schreiber S.L. (2003). Domain-selective small-molecule inhibitor of histone deacetylase 6 (HDAC6)-mediated tubulin deacetylation. Proc. Natl. Acad. Sci. USA.

[17] Butler K.V., Kalin J., Brochier C., Vistoli G., Langley B., Kozikowski A.P. (2010). Rational design and simple chemistry yield a superior, neuroprotective HDAC6 inhibitor, tubastatin A. J. Am. Chem. Soc..

[18] Mithraprabhu S., Khong T., Jones S.S., Spencer A. (2013). Histone deacetylase (HDAC) inhibitors as single agents induce multiple myeloma cell death principally through the inhibition of class I HDAC. Br. J. Haematol..

[19] Santo L., Hideshima T., Kung A.L., Tseng J.C., Tamang D., Yang M., Jarpe M., van Duzer J.H., Mazitschek R., Ogier W.C. (2012). Preclinical activity, pharmacodynamic, and pharmacokinetic properties of a selective HDAC6 inhibitor, ACY-1215, in combination with bortezomib in multiple myeloma. Blood.

[20] Strebl M.G., Campbell A.J., Zhao W.N., Schroeder F.A., Riley M.M., Chindavong P.S., Morin T.M., Haggarty S.J., Wagner F.F., Ritter T. (2017). HDAC6 Brain Mapping with [^18^F]Bavarostat Enabled by a Ru-Mediated Deoxyfluorination. ACS Cent. Sci..

[21] Balasubramanian S., Ramos J., Luo W., Sirisawad M., Verner E., Buggy J.J. (2008). A novel histone deacetylase 8 (HDAC8)-specific inhibitor PCI-34051 induces apoptosis in T-cell lymphomas. Leukemia.

[22] Jochems J., Boulden J., Lee B.G., Blendy J.A., Jarpe M., Mazitschek R., Van Duzer J.H., Jones S., Berton O. (2014). Antidepressant-like properties of novel HDAC6-selective inhibitors with improved brain bioavailability. Neuropsychopharmacology.

[23] Majid T., Griffin D., Criss Z., Jarpe M., Pautler R.G. (2015). Pharmocologic treatment with histone deacetylase 6 inhibitor (ACY-738) recovers Alzheimer’s disease phenotype in amyloid precursor protein/presenilin 1 (APP/PS1) mice. Alzheimer’s Dement..

[24] Rivieccio M.A., Brochier C., Willis D.E., Walker B.A., D’Annibale M.A., McLaughlin K., Siddiq A., Kozikowski A.P., Jaffrey S.R., Twiss J.L. (2009). HDAC6 is a target for protection and regeneration following injury in the nervous system. Proc. Natl. Acad. Sci. USA.

[25] Hanson K., Tian N., Vickers J.C., King A.E. (2018). The HDAC6 Inhibitor Trichostatin A Acetylates Microtubules and Protects Axons from Excitotoxin-Induced Degeneration in a Compartmented Culture Model. Front. Neurosci..

[26] Ganai S.A. (2017). Small-molecule Modulation of HDAC6 Activity: The Propitious Therapeutic Strategy to Vanquish Neurodegenerative Disorders. Curr. Med. Chem..

[27] Kim S.H., Shanware N.P., Bowler M.J., Tibbetts R.S. (2010). Amyotrophic lateral sclerosis-associated proteins TDP-43 and FUS/TLS function in a common biochemical complex to co-regulate HDAC6 mRNA. J. Biol. Chem..

[28] Fiesel F.C., Voigt A., Weber S.S., Van den Haute C., Waldenmaier A., Görner K., Walter M., Anderson M.L., Kern J.V., Rasse T.M. (2010). Knockdown of transactive response DNA-binding protein (TDP-43) downregulates histone deacetylase 6. EMBO J..

[29] Miskiewicz K., Jose L.E., Yeshaw W.M., Valadas J.S., Swerts J., Munck S., Feiguin F., Dermaut B., Verstreken P. (2014). HDAC6 is a Bruchpilot deacetylase that facilitates neurotransmitter release. Cell Rep..

[30] Xia Q., Wang H., Zhang Y., Ying Z., Wang G. (2015). Loss of TDP-43 Inhibits Amyotrophic Lateral Sclerosis-Linked Mutant SOD1 Aggresome Formation in an HDAC6-Dependent Manner. J. Alzheimer’s Dis..

[31] Chen S., Zhang X.J., Li L.X., Wang Y., Zhong R.J., Le W. (2015). Histone deacetylase 6 delays motor neuron degeneration by ameliorating the autophagic flux defect in a transgenic mouse model of amyotrophic lateral sclerosis. Neurosci. Bull..

[32] Gal J., Chen J., Barnett K.R., Yang L., Brumley E., Zhu H. (2013). HDAC6 regulates mutant SOD1 aggregation through two SMIR motifs and tubulin acetylation. J. Biol. Chem..

[33] Lee J.Y., Kawaguchi Y., Li M., Kapur M., Choi S.J., Kim H.J., Park S.Y., Zhu H., Yao T.P. (2015). Uncoupling of Protein Aggregation and Neurodegeneration in a Mouse Amyotrophic Lateral Sclerosis Model. Neurodegener. Dis..

[34] Taes I., Timmers M., Hersmus N., Bento-Abreu A., Van Den Bosch L., Van Damme P., Auwerx J., Robberecht W. (2013). Hdac6 deletion delays disease progression in the SOD1G93A mouse model of ALS. Hum. Mol. Genet..

[35] Fitzgerald K. (2005). RNAi versus small molecules: Different mechanisms and specificities can lead to different outcomes. Curr. Opin. Drug Discov. Dev..

[36] Weiss W.A., Taylor S.S., Shokat K.M. (2007). Recognizing and exploiting differences between RNAi and small-molecule inhibitors. Nat. Chem. Biol..

[37] Tseng J.H., Xie L., Song S., Xie Y., Allen L., Ajit D., Hong J.S., Chen X., Meeker R.B., Cohen T.J. (2017). The Deacetylase HDAC6 Mediates Endogenous Neuritic Tau Pathology. Cell Rep..

[38] Govindarajan N., Rao P., Burkhardt S., Sananbenesi F., Schlüter O.M., Bradke F., Lu J., Fischer A. (2013). Reducing HDAC6 ameliorates cognitive deficits in a mouse model for Alzheimer’s disease. EMBO Mol. Med..

[39] Selenica M.L., Benner L., Housley S.B., Manchec B., Lee D.C., Nash K.R., Kalin J., Bergman J.A., Kozikowski A., Gordon M.N. (2014). Histone deacetylase 6 inhibition improves memory and reduces total tau levels in a mouse model of tau deposition. Alzheimer’s Res..

[40] Zhang L., Liu C., Wu J., Tao J.J., Sui X.L., Yao Z.G., Xu Y.F., Huang L., Zhu H., Sheng S.L. (2014). Tubastatin A/ACY-1215 Improves Cognition in Alzheimer’s Disease Transgenic Mice. J. Alzheimer’s Dis..

[41] Pietropaolo S., Feldon J., Yee B.K. (2008). Age-dependent phenotypic characteristics of a triple transgenic mouse model of Alzheimer disease. Behav. Neurosci..

[42] Fan S.J., Huang F.I., Liou J.P., Yang C.R. (2018). The novel histone de acetylase 6 inhibitor, MPT0G211, ameliorates tau phosphorylation and cognitive deficits in an Alzheimer’s disease model. Cell Death Dis..

[43] Benoy V., Van Helleputte L., Prior R., d’Ydewalle C., Haeck W., Geens N., Scheveneels W., Schevenels B., Cader M.Z., Talbot K. (2018). HDAC6 is a therapeutic target in mutant GARS-induced Charcot-Marie-Tooth disease. Brain.

[44] Shen S., Benoy V., Bergman J.A., Kalin J.H., Frojuello M., Vistoli G., Haeck W., Van Den Bosch L., Kozikowski A.P. (2016). Bicyclic-Capped Histone Deacetylase 6 Inhibitors with Improved Activity in a Model of Axonal Charcot-Marie-Tooth Disease. ACS Chem. Neurosci..

[45] Prior R., Van Helleputte L., Klingl Y.E., Van Den Bosch L. (2018). HDAC6 as a potential therapeutic target for peripheral nerve disorders. Expert Opin. Targets.

[46] Benoy V., Vanden Berghe P., Jarpe M., Van Damme P., Robberecht W., Van Den Bosch L. (2017). Development of Improved HDAC6 Inhibitors as Pharmacological Therapy for Axonal Charcot-Marie-Tooth Disease. Neurotherapeutics.

[47] Mo Z., Zhao X., Liu H., Hu Q., Chen X.Q., Pham J., Wei N., Liu Z., Zhou J., Burgess R.W. (2018). Aberrant GlyRS-HDAC6 interaction linked to axonal transport deficits in Charcot-Marie-Tooth neuropathy. Nat. Commun..

[48] Gold W.A., Lacina T.A., Cantrill L.C., Christodoulou J. (2015). MeCP2 deficiency is associated with reduced levels of tubulin acetylation and can be restored using HDAC6 inhibitors. J. Mol. Med..

[49] Richter-Landsberg C. (2016). Protein aggregate formation in oligodendrocytes: Tau and the cytoskeleton at the intersection of neuroprotection and neurodegeneration. Biol. Chem..

[50] Richter-Landsberg C., Leyk J. (2013). Inclusion body formation, macroautophagy, and the role of HDAC6 in neurodegeneration. Acta Neuropathol..

[51] Noack M., Leyk J., Richter-Landsberg C. (2014). HDAC6 inhibition results in tau acetylation and modulates tau phosphorylation and degradation in oligodendrocytes. Glia.

[52] Leyk J., Goldbaum O., Noack M., Richter-Landsberg C. (2015). Inhibition of HDAC6 modifies tau inclusion body formation and impairs autophagic clearance. J. Mol. Neurosci..

[53] Leyk J., Daly C., Janssen-Bienhold U., Kennedy B.N., Richter-Landsberg C. (2017). HDAC6 inhibition by tubastatin A is protective against oxidative stress in a photoreceptor cell line and restores visual function in a zebrafish model of inherited blindness. Cell Death Dis..

[54] Smith M.A., Evans S.M. (2017). Introduction to special issue on animal models of neuropsychiatric disorders and substance use disorders: Progress and gaps. Exp. Clin. Psychopharmacol..

[55] Kaiser T., Zhou Y., Feng G. (2017). Animal models for neuropsychiatric disorders: Prospects for circuit intervention. Curr. Opin. Neurobiol..

[56] Pound P., Ebrahim S., Sandercock P., Bracken M.B., Roberts I. (2004). Where is the evidence that animal research benefits humans?. BMJ.

[57] Hackam D.G., Redelmeier D.A. (2006). Translation of research evidence from animals to humans. JAMA.

[58] Medicine I.O. (2013). Improving the Utility and Translation of Animal Models for Nervous System Disorders: Workshop Summary.

[59] Akhtar A. (2015). The flaws and human harms of animal experimentation. Camb. Q. Healthc. Ethics.

[60] Lal S., Li A., Dos Remedios C. (2016). Limitations in Translating Animal Studies to Humans in Cardiovascular Disease. J. Cardiovasc. Transl. Res..

[61] Seok J., Warren H.S., Cuenca A.G., Mindrinos M.N., Baker H.V., Xu W., Richards D.R., McDonald-Smith G.P., Gao H., Hennessy L. (2013). Genomic responses in mouse models poorly mimic human inflammatory diseases. Proc. Natl. Acad. Sci. USA.

[62] Pak C., Danko T., Zhang Y., Aoto J., Anderson G., Maxeiner S., Yi F., Wernig M., Südhof T.C. (2015). Human Neuropsychiatric Disease Modeling using Conditional Deletion Reveals Synaptic Transmission Defects Caused By Heterozygous Mutations in NRXN1. Cell Stem Cell.

[63] van der Worp H.B., Howells D.W., Sena E.S., Porritt M.J., Rewell S., O’Collins V., Macleod M.R. (2010). Can animal models of disease reliably inform human studies?. PLoS Med..

[64] Rice J. (2012). Animal models: Not close enough. Nature.

[65] Huang J.H., Berkovitch S.S., Iaconelli J., Watmuff B., Park H., Chattopadhyay S., McPhie D., Öngür D., Cohen B.M., Clish C.B. (2016). Perturbational Profiling of Metabolites in Patient Fibroblasts Implicates α-Aminoadipate as a Potential Biomarker for Bipolar Disorder. Mol. Neuropsychiatry.

[66] Huang J.H., Park H., Iaconelli J., Berkovitch S.S., Watmuff B., McPhie D., Öngür D., Cohen B.M., Clish C.B., Karmacharya R. (2017). Unbiased Metabolite Profiling of Schizophrenia Fibroblasts under Stressful Perturbations Reveals Dysregulation of Plasmalogens and Phosphatidylcholines. J. Proteome Res..

[67] Wimalasena N.K., Le V.Q., Wimalasena K., Schreiber S.L., Karmacharya R. (2016). Gene Expression-Based Screen for Parkinson’s Disease Identifies GW8510 as a Neuroprotective Agent. ACS Chem. Neurosci..

[68] Takahashi K., Tanabe K., Ohnuki M., Narita M., Ichisaka T., Tomoda K., Yamanaka S. (2007). Induction of pluripotent stem cells from adult human fibroblasts by defined factors. Cell.

[69] Shi Y., Inoue H., Wu J.C., Yamanaka S. (2017). Induced pluripotent stem cell technology: A decade of progress. Nat. Rev. Drug Discov..

[70] Karmacharya R., Haggarty S.J. (2016). Stem cell models of neuropsychiatric disorders. Mol. Cell. Neurosci..

[71] Watmuff B., Liu B., Karmacharya R. (2017). Stem cell-derived neurons in the development of targeted treatment for schizophrenia and bipolar disorder. Pharmacogenomics.

[72] Watmuff B., Berkovitch S.S., Huang J.H., Iaconelli J., Toffel S., Karmacharya R. (2016). Disease signatures for schizophrenia and bipolar disorder using patient-derived induced pluripotent stem cells. Mol. Cell. Neurosci..

[73] Berkovitch S.S., Iaconelli J., Karmacharya R. (2015). Patient-Derived iPSCs as a Model for Schizophrenia. J. Stem Cell Res. Regener. Med..

[74] Kirwan P., Turner-Bridger B., Peter M., Momoh A., Arambepola D., Robinson H.P., Livesey F.J. (2015). Development and function of human cerebral cortex neural networks from pluripotent stem cells in vitro. Development.

[75] Kim J.Y., Woo S.Y., Hong Y.B., Choi H., Kim J., Mook-Jung I., Ha N., Kyung J., Koo S.K., Jung S.C. (2016). HDAC6 Inhibitors Rescued the Defective Axonal Mitochondrial Movement in Motor Neurons Derived from the Induced Pluripotent Stem Cells of Peripheral Neuropathy Patients with. Stem Cells Int..

[76] Guo W., Naujock M., Fumagalli L., Vandoorne T., Baatsen P., Boon R., Ordovás L., Patel A., Welters M., Vanwelden T. (2017). HDAC6 inhibition reverses axonal transport defects in motor neurons derived from FUS-ALS patients. Nat. Commun..

[77] Landucci E., Brindisi M., Bianciardi L., Catania L.M., Daga S., Croci S., Frullanti E., Fallerini C., Butini S., Brogi S. (2018). iPSC-derived neurons profiling reveals GABAergic circuit disruption and acetylated α-tubulin defect which improves after iHDAC6 treatment in Rett syndrome. Exp. Cell Res..

[78] Misztak P., Pańczyszyn-Trzewik P., Sowa-Kućma M. (2018). Histone deacetylases (HDACs) as therapeutic target for depressive disorders. Pharm. Rep..

[79] Machado-Vieira R., Ibrahim L., Zarate C.A. (2011). Histone deacetylases and mood disorders: Epigenetic programming in gene-environment interactions. CNS Neurosci..

[80] Erburu M., Muñoz-Cobo I., Domínguez-Andrés J., Beltran E., Suzuki T., Mai A., Valente S., Puerta E., Tordera R.M. (2015). Chronic stress and antidepressant induced changes in Hdac5 and Sirt2 affect synaptic plasticity. Eur. Neuropsychopharmacol..

[81] Phiel C.J., Zhang F., Huang E.Y., Guenther M.G., Lazar M.A., Klein P.S. (2001). Histone deacetylase is a direct target of valproic acid, a potent anticonvulsant, mood stabilizer, and teratogen. J. Biol. Chem..

[82] Covington H.E., Maze I., Vialou V., Nestler E.J. (2015). Antidepressant action of HDAC inhibition in the prefrontal cortex. Neuroscience.

[83] Schroeder F.A., Lewis M.C., Fass D.M., Wagner F.F., Zhang Y.L., Hennig K.M., Gale J., Zhao W.N., Reis S., Barker D.D. (2013). A selective HDAC 1/2 inhibitor modulates chromatin and gene expression in brain and alters mouse behavior in two mood-related tests. PLoS ONE.

[84] Meylan E.M., Halfon O., Magistretti P.J., Cardinaux J.R. (2016). The HDAC inhibitor SAHA improves depressive-like behavior of CRTC1-deficient mice: Possible relevance for treatment-resistant depression. Neuropharmacology.

[85] Sun J., Wang F., Hong G., Pang M., Xu H., Li H., Tian F., Fang R., Yao Y., Liu J. (2016). Antidepressant-like effects of sodium butyrate and its possible mechanisms of action in mice exposed to chronic unpredictable mild stress. Neurosci. Lett..

[86] Golden S.A., Christoffel D.J., Heshmati M., Hodes G.E., Magida J., Davis K., Cahill M.E., Dias C., Ribeiro E., Ables J.L. (2013). Epigenetic regulation of RAC1 induces synaptic remodeling in stress disorders and depression. Nat. Med..

[87] Fukada M., Hanai A., Nakayama A., Suzuki T., Miyata N., Rodriguiz R.M., Wetsel W.C., Yao T.P., Kawaguchi Y. (2012). Loss of deacetylation activity of Hdac6 affects emotional behavior in mice. PLoS ONE.

[88] Maguschak K.A., Ressler K.J. (2012). The dynamic role of beta-catenin in synaptic plasticity. Neuropharmacology.

[89] Gould T.D., Einat H., O’Donnell K.C., Picchini A.M., Schloesser R.J., Manji H.K. (2007). Beta-catenin overexpression in the mouse brain phenocopies lithium-sensitive behaviors. Neuropsychopharmacology.

[90] Gould T.D., O’Donnell K.C., Picchini A.M., Dow E.R., Chen G., Manji H.K. (2008). Generation and behavioral characterization of beta-catenin forebrain-specific conditional knock-out mice. Behav. Brain Res..

[91] Valvezan A.J., Klein P.S. (2012). GSK-3 and Wnt Signaling in Neurogenesis and Bipolar Disorder. Front. Mol. Neurosci..

[92] Dias C., Feng J., Sun H., Shao N.Y., Mazei-Robison M.S., Damez-Werno D., Scobie K., Bagot R., LaBonte B., Ribeiro E. (2014). beta-catenin mediates stress resilience through Dicer1/microRNA regulation. Nature.

[93] Mulligan K.A., Cheyette B.N. (2017). Neurodevelopmental Perspectives on Wnt Signaling in Psychiatry. Mol. Neuropsychiatry.

[94] Li X., Jope R.S. (2010). Is glycogen synthase kinase-3 a central modulator in mood regulation?. Neuropsychopharmacology.

[95] Karege F., Perroud N., Burkhardt S., Fernandez R., Ballmann E., La Harpe R., Malafosse A. (2012). Protein levels of β-catenin and activation state of glycogen synthase kinase-3β in major depression. A study with postmortem prefrontal cortex. J. Affect. Disord..

[96] Gould T.D., Dow E.R., O’Donnell K.C., Chen G., Manji H.K. (2007). Targeting signal transduction pathways in the treatment of mood disorders: Recent insights into the relevance of the Wnt pathway. CNS Neurol. Disord. Drug Targets.

[97] O’Donnell K.C., Gould T.D. (2007). The behavioral actions of lithium in rodent models: Leads to develop novel therapeutics. Neurosci. Biobehav. Rev..

[98] Bersudsky Y., Shaldubina A., Belmaker R.H. (2007). Lithium’s effect in forced-swim test is blood level dependent but not dependent on weight loss. Behav Pharm..

[99] O’Brien W.T., Harper A.D., Jove F., Woodgett J.R., Maretto S., Piccolo S., Klein P.S. (2004). Glycogen synthase kinase-3beta haploinsufficiency mimics the behavioral and molecular effects of lithium. J. Neurosci..

[100] Valenta T., Hausmann G., Basler K. (2012). The many faces and functions of beta-catenin. EMBO J..

[101] Bielen H., Houart C. (2014). The Wnt cries many: Wnt regulation of neurogenesis through tissue patterning, proliferation, and asymmetric cell division. Dev. Neurobiol..

[102] Nusse R., Clevers H. (2017). Wnt/β-Catenin Signaling, Disease, and Emerging Therapeutic Modalities. Cell.

[103] Iaconelli J., Huang J.H., Berkovitch S.S., Chattopadhyay S., Mazitschek R., Schreiber S.L., Haggarty S.J., Karmacharya R. (2015). HDAC6 inhibitors modulate Lys49 acetylation and membrane localization of beta-catenin in human iPSC-derived neuronal cells. ACS Chem. Boil..

[104] Wolf D., Rodova M., Miska E.A., Calvet J.P., Kouzarides T. (2002). Acetylation of beta-catenin by CREB-binding protein (CBP). J. Boil. Chem..

[105] Garcia-Rostan G., Tallini G., Herrero A., D’Aquila T.G., Carcangiu M.L., Rimm D.L. (1999). Frequent mutation and nuclear localization of beta-catenin in anaplastic thyroid carcinoma. Cancer Res..

[106] Li Y., Zhang X., Polakiewicz R.D., Yao T.P., Comb M.J. (2008). HDAC6 is required for epidermal growth factor-induced beta-catenin nuclear localization. J. Boil. Chem..

[107] Jiang S., Zhang M., Sun J., Yang X. (2018). Casein kinase 1α: Biological mechanisms and theranostic potential. Cell Commun. Signal..

[108] Amit S., Hatzubai A., Birman Y., Andersen J.S., Ben-Shushan E., Mann M., Ben-Neriah Y., Alkalay I. (2002). Axin-mediated CKI phosphorylation of beta-catenin at Ser 45: A molecular switch for the Wnt pathway. Genes Dev..

[109] Maher M.T., Mo R., Flozak A.S., Peled O.N., Gottardi C.J. (2010). Beta-catenin phosphorylated at serine 45 is spatially uncoupled from beta-catenin phosphorylated in the GSK3 domain: Implications for signaling. PLoS ONE.

[110] Winer I.S., Bommer G.T., Gonik N., Fearon E.R. (2006). Lysine residues Lys-19 and Lys-49 of beta-catenin regulate its levels and function in T cell factor transcriptional activation and neoplastic transformation. J. Boil. Chem..

[111] Caron C., Boyault C., Khochbin S. (2005). Regulatory cross-talk between lysine acetylation and ubiquitination: Role in the control of protein stability. BioEssays.

[112] Nihira N.T., Ogura K., Shimizu K., North B.J., Zhang J., Gao D., Inuzuka H., Wei W. (2017). Acetylation-dependent regulation of MDM2 E3 ligase activity dictates its oncogenic function. Sci. Signal..

[113] Lalonde J., Reis S.A., Sivakumaran S., Holland C.S., Wesseling H., Sauld J.F., Alural B., Zhao W.N., Steen J.A., Haggarty S.J. (2017). Chemogenomic analysis reveals key role for lysine acetylation in regulating Arc stability. Nat. Commun..

[114] Min S.W., Cho S.H., Zhou Y., Schroeder S., Haroutunian V., Seeley W.W., Huang E.J., Shen Y., Masliah E., Mukherjee C. (2010). Acetylation of tau inhibits its degradation and contributes to tauopathy. Neuron.

[115] Ge X., Jin Q., Zhang F., Yan T., Zhai Q. (2009). PCAF acetylates {beta}-catenin and improves its stability. Mol. Biol. Cell..

[116] Seong E., Yuan L., Arikkath J. (2015). Cadherins and catenins in dendrite and synapse morphogenesis. Cell Adhes. Migr..

[117] Ochs S.M., Dorostkar M.M., Aramuni G., Schön C., Filser S., Pöschl J., Kremer A., Van Leuven F., Ovsepian S.V., Herms J. (2015). Loss of neuronal GSK3β reduces dendritic spine stability and attenuates excitatory synaptic transmission via β-catenin. Mol. Psychiatry.

[118] Bian W.J., Miao W.Y., He S.J., Qiu Z., Yu X. (2015). Coordinated Spine Pruning and Maturation Mediated by Inter-Spine Competition for Cadherin/Catenin Complexes. Cell.

[119] Peng Y.R., He S., Marie H., Zeng S.Y., Ma J., Tan Z.J., Lee S.Y., Malenka R.C., Yu X. (2009). Coordinated changes in dendritic arborization and synaptic strength during neural circuit development. Neuron.

[120] Beurel E., Grieco S.F., Amadei C., Downey K., Jope R.S. (2016). Ketamine-induced inhibition of glycogen synthase kinase-3 contributes to the augmentation of α-amino-3-hydroxy-5-methylisoxazole-4-propionic acid (AMPA) receptor signaling. Bipolar Disord..

[121] Liu R.J., Fuchikami M., Dwyer J.M., Lepack A.E., Duman R.S., Aghajanian G.K. (2013). GSK-3 inhibition potentiates the synaptogenic and antidepressant-like effects of subthreshold doses of ketamine. Neuropsychopharmacology.

[122] Pilar-Cuellar F., Vidal R., Pazos A. (2012). Subchronic treatment with fluoxetine and ketanserin increases hippocampal brain-derived neurotrophic factor, beta-catenin and antidepressant-like effects. Br. J. Pharmacol..

[123] Madsen T.M., Newton S.S., Eaton M.E., Russell D.S., Duman R.S. (2003). Chronic electroconvulsive seizure up-regulates β-catenin expression in rat hippocampus: Role in adult neurogenesis. Biol. Psychiatry.

[124] Kim E., Sheng M. (2004). PDZ domain proteins of synapses. Nat. Rev. Neurosci..

[125] Toto A., Pedersen S.W., Karlsson O.A., Moran G.E., Andersson E., Chi C.N., Strømgaard K., Gianni S., Jemth P. (2016). Ligand binding to the PDZ domains of postsynaptic density protein 95. Protein Eng. Des. Sel..

[126] Manjunath G.P., Ramanujam P.L., Galande S. (2018). Structure function relations in PDZ-domain-containing proteins: Implications for protein networks in cellular signalling. J. Biosci..

[127] Gujral T.S., Karp E.S., Chan M., Chang B.H., MacBeath G. (2013). Family-wide investigation of PDZ domain-mediated protein-protein interactions implicates beta-catenin in maintaining the integrity of tight junctions. Chem. Biol..

[128] Iaconelli J., Lalonde J., Watmuff B., Liu B., Mazitschek R., Haggarty S.J., Karmacharya R. (2017). Lysine Deacetylation by HDAC6 Regulates the Kinase Activity of AKT in Human Neural Progenitor Cells. ACS Chem. Boil..

[129] Jochems J., Teegarden S.L., Chen Y., Boulden J., Challis C., Ben-Dor G.A., Kim S.F., Berton O. (2015). Enhancement of stress resilience through histone deacetylase 6-mediated regulation of glucocorticoid receptor chaperone dynamics. Biol. Psychiatry.

[130] Karmacharya R., Sliwoski G.R., Lundy M.Y., Suckow R.F., Cohen B.M., Buttner E.A. (2009). Clozapine interaction with phosphatidyl inositol 3-kinase (PI3K)/insulin-signaling pathway in Caenorhabditis elegans. Neuropsychopharmacology.

[131] Del’Guidice T., Beaulieu J.M. (2015). Selective disruption of dopamine D2-receptors/beta-arrestin2 signaling by mood stabilizers. J. Recept. Signal Transduct. Res..

[132] Gao Y., Peterson S., Masri B., Hougland M.T., Adham N., Gyertyán I., Kiss B., Caron M.G., El-Mallakh R.S. (2015). Cariprazine exerts antimanic properties and interferes with dopamine D2 receptor β-arrestin interactions. Pharmacol. Res. Perspect..

[133] Majumdar D., Nebhan C.A., Hu L., Anderson B., Webb D.J. (2011). An APPL1/Akt signaling complex regulates dendritic spine and synapse formation in hippocampal neurons. Mol. Cell. Neurosci..

[134] Pillai V.B., Sundaresan N.R., Gupta M.P. (2014). Regulation of Akt signaling by sirtuins: Its implication in cardiac hypertrophy and aging. Circ. Res..

[135] Ramakrishnan G., Davaakhuu G., Kaplun L., Chung W.C., Rana A., Atfi A., Miele L., Tzivion G. (2014). Sirt2 deacetylase is a novel AKT binding partner critical for AKT activation by insulin. J. Boil. Chem..

[136] Zhang J., Shemezis J.R., McQuinn E.R., Wang J., Sverdlov M., Chenn A. (2013). AKT activation by N-cadherin regulates beta-catenin signaling and neuronal differentiation during cortical development. Neural Dev..

[137] Jansen L.A., Mirzaa G.M., Ishak G.E., O’Roak B.J., Hiatt J.B., Roden W.H., Gunter S.A., Christian S.L., Collins S., Adams C. (2015). PI3K/AKT pathway mutations cause a spectrum of brain malformations from megalencephaly to focal cortical dysplasia. Brain.

[138] Chu W., Yuan J., Huang L., Xiang X., Zhu H., Chen F., Chen Y., Lin J., Feng H. (2015). Valproic Acid Arrests Proliferation but Promotes Neuronal Differentiation of Adult Spinal NSPCs from SCI Rats. Neurochem. Res..

